# “Every Tooth Containing a Metallic Filling Is a Galvanic Battery in Active Operation,” Etc.

**Published:** 1880-02

**Authors:** Henry S. Chase

**Affiliations:** St. Louis, Mo.


ARTICLE IV.

### "EVERY TOOTH CONTAINING A METALLIC FILLING IS A GALVANIC BATTERY IN ACTIVE OPERATION WHEN THE SURROUNDING FLUID HAS AN ACID REACTION."

BY HENRY S. CHASE, M. D., D. D. S., ST. LOUIS, MO.

That the title of this article is true, no "professor of physical science" will deny; and every dentist, who has studied the elements of electrical science, must admit that the proposition is in harmony with the principles of electricity as set forth in the text books on that subject. One would suppose that after the active warfare of three years against the principles of the "new departure," that a large number of dentists would have applied themselves to learning, at least, the elements of the science of galvanism and electricity; but the utter ignorance of this subject displayed in the productions of nearly all the writers against the "new departure" is truly lamentable. A galvanic battery may be composed of almost any of two different substances. They need not even be metals. It is only necessary that one of them can be more easily acted upon chemically than the other. Galvanic action promotes chemical action.

Example: Place a small copper strip in a glass vessel. Place a zinc strip of the same size in a similar vessel. Pour into each the same quantity of diluted vinegar or lemon juice. After an hour's immersion each will have lost something in weight. Now place the zinc and copper in the same vessel, allowing the bottom ends to come in contact. After one hour's immersion it will be found that the zinc has lost more in weight and the copper less in weight than when each metal was by itself. The cause is increased chemical action, stimulated by the production of the electric current. An electric current is easily established. Hardly any two substances can come in contact without producing a current of electricity.

Example 2: Place a strip of gold in a vessel. Place a strip of pure tin in another vessel. Pour into each vessel a quantity of vinegar. After an hour the gold will not have lost anything in weight. The tin will have lost a definite amount. Place them both in the same vessel and liquid, with their lower ends touching, for an hour. At the end of that time the tin will be found to have lost more than when in the fluid by itself.

The best conductors of electricity form the strongest or most intense batteries. The stronger the battery the more the positive element will lose in weight. The strongest battery is where the negative element will not lose any weight, and where the positive will lose weight rapidly. Let us apply these principles to the substances with which dentists have to do. Gold is the best conductor, tin next, and amalgams next. Value of each:

| | |
|---|---|
| Gold . . . . . . . . . . . . | 60 |
| Tin . . . . . . . . . . . . | 30 |
| Amalgams . . . . . . . . . . | 10 |

A gold plug in a tooth forms the elements of a strong battery, because the gold will not dissolve in acidulous saliva and dentos will. Amalgams and dentos make a battery of less strength. Tin and dentos form a still weaker battery. These metals are all negative to dentos. Dentos is positive to all these metals. The most negative and the most positive substances form the strongest batteries, other conditions being the same. Amalgams form a stronger battery with dentos than tin, because they are more negative to dentos than tin. We have just seen that amalgams are poorer conductors than tin, and consequently if they were only as negative to dentos as tin they would make a far weaker battery with dentos than tin. The strength of the batteries is as follows:

| | |
|---|---|
| Call Gold and Dentos . . . . . . . . | 100 |
| Then Amalgams and Dentos . . . . . . | 67 |
| Tin and Dentos . . . . . . . . . . . | 50 |

In example 1 we saw that the copper or negative element when in contact with the zinc lost less than when it stood alone. It is found that the positive element always suffers more, and the negative element less, in a battery than the same elements would disconnected.

Example 3: Take a plate of dental amalgam and a plate of dentine, and place them apart in a glass of dilute acid for a month. At the end of that time each will have lost something in weight. Now place their lower ends together in the same strength fluid, and let them remain another month. At the end of that time the amalgam plate will have lost less in weight and the dentine more in weight than they did when standing without contact. We have seen that the element that is most easily dissolved is called the *positive* element. Dentos is positive to gold, amalgam and tin. Consequently, dentos is more rapidly dissolved when connected with those elements than when alone. But dentos is not positive to all filling materials. Dentos is *negative* to ox. phos. zinc. Ox. phos. zinc is positive to dentos. When these are united in battery the dentos loses less and the ox. phos. zinc more than when not in contact in the same fluid. The ox. chlo. zinc is also positive to dentos.

Some cubes of dentine were prepared six m. square, with a hole through the cube four m. diameter. The hole in one was filled with each of the following:

Pure gold, dental amalgam, tin foil, gutta percha, ox. chlo. zinc, wax.

They were placed in a large, shallow plate, and covered with a weak mixture of vinegar and water. The cubes previous to being plugged were brought to exactly the same weight in c. g. After one week's immersion the fillings were removed one by one and weighed. The empty cubes lost in weight as follows:

| | |
|---|---|
| Gold Filled Cube . . . . . . . . | 6 c. g. |
| Amalgam Filled Cube . . . . . . . | 4 c. g. |
| Tin " " . . . . . . . . | 3 c. g. |
| Gutta Percha Filled Cube . . . . . . | 1 c. g. |
| Wax " " . . . . . . | 1 c. g. |
| Ox. Chlo. Zinc " " . . . . . . | 0 c. g. |

These experiments accord exactly with the principles of electrical science. The cube losing nothing was negative to the ox. chlo. zinc, and, therefore, that filling lost something of its own weight while *preserving* the cube. The same experiments were tried with weak lemon juice, with like results. There is nothing in living dentine to prevent these same phenomena from occurring in the mouth. The conditions are even more favorable, for living dentos is a better conductor of electricity than dead dentine, and with better conductivity the phenomena would be intensified.

*In the Mouth.*—Gold fillings on the grinding surfaces of teeth have a longer existence than in any other locality. These surfaces are less likely to be bathed in acidulous fluids. Gold fillings on the approximal surfaces of teeth near the gums have the shortest existence. In the latter case they are subject to acid surroundings from various causes. Leaky gold plugs are of short duration, owing to galvanic action within the cavity, and thus the plug commits suicide. A leaky amalgam plug rusts, and the oxide of tin and silver is formed, which soaks into the dentine and preserves it by making the dentine less positive to the filling, and thus the galvanic action, etc., ceases. These oxides take up more room than the metals from which they were formed, and thus the leakage is in a measure stopped. No such recuperative action is possible under a gold plug. Gutta percha fillings being non-conductors form no batteries with dentos. If one leaks there is no intensified chemical action as in the former cases. The ox. chlo. zinc and the ox. phos. zinc fillings form weak batteries in the teeth, which latter are negative to the plugs. Consequently, the teeth are preserved at the expense of the plugs. At the edge of the gum, in proximate cavities, these plugs soon dissolve, but the dentine is preserved as long as it is covered with the filling. Had a gold filling been here the dentine would have dissolved under the same conditions. If gutta percha is placed at the cervical border of such a cavity, and the remainder filled with an ox. phos. zinc, the battery will be weakened, and the ox. phos. last longer.

Do we wish to preserve the teeth that we fill, or had we rather preserve the fillings themselves? We remove a gold plug from a cavity, the dentine of which has decayed under the filling. The plug is whole, but is useless, except as old gold. The cavity must be excavated again and replugged. Suppose we do this with a filling which will itself wear out while preserving the tooth. Is it not better to preserve a tooth than a plug? Let us always keep in mind the object of our profession: the preservation and health of the teeth.

